# Standardized Integration of Person-Generated Data Into Routine Clinical Care

**DOI:** 10.2196/31048

**Published:** 2022-02-10

**Authors:** Billy Zeng, Riley Bove, Simona Carini, Jonathan Shing-Jih Lee, JP Pollak, Erica Schleimer, Ida Sim

**Affiliations:** 1 Division of General Internal Medicine University of California, San Francisco San Francisco, CA United States; 2 University of California, San Francisco Weill Institute for Neurosciences Department of Neurology University of California, San Francisco San Francisco, CA United States; 3 The Commons Project New York, NY United States

**Keywords:** mobile health, data sharing, health care, patient-generated health data, telemedicine

## Abstract

Person-generated data (PGD) are a valuable source of information on a person’s health state in daily life and in between clinic visits. To fully extract value from PGD, health care organizations must be able to smoothly integrate data from PGD devices into routine clinical workflows. Ideally, to enhance efficiency and flexibility, such integrations should follow reusable processes that can easily be replicated for multiple devices and data types. Instead, current PGD integrations tend to be one-off efforts entailing high costs to build and maintain custom connections with each device and their proprietary data formats. This viewpoint paper formulates the integration of PGD into clinical systems and workflow as a *PGD integration pipeline* and reviews the functional components of such a pipeline. A PGD integration pipeline includes PGD acquisition, aggregation, and consumption. Acquisition is the person-facing component that includes both technical (eg, sensors, smartphone apps) and policy components (eg, informed consent). Aggregation pools, standardizes, and structures data into formats that can be used in health care settings such as within electronic health record–based workflows. PGD consumption is wide-ranging, by different solutions in different care settings (inpatient, outpatient, consumer health) for different types of users (clinicians, patients). The adoption of data and metadata standards, such as those from IEEE and Open mHealth, would facilitate aggregation and enable broader consumption. We illustrate the benefits of a standards-based integration pipeline for the illustrative use case of home blood pressure monitoring. A standards-based PGD integration pipeline can flexibly streamline the clinical use of PGD while accommodating the complexity, scale, and rapid evolution of today’s health care systems.

## Introduction

Person-generated data (PGD) are a valuable source of information on a person’s health state in daily life and in between clinic visits [1]. PGD can be acquired via apps, sensors, wearables, or simple online forms, which we will collectively call *PGD devices*.

To fully extract value from PGD, health care organizations must be able to smoothly integrate data from PGD devices into routine clinical workflows. For example, in an ideal remote blood pressure (BP) monitoring program, clinicians will “prescribe” a BP monitoring plan (eg, measure BP every morning for the next 2 weeks). The patient will collect and share BP data from their Bluetooth-connected wireless cuff, data that will be seamlessly integrated into the clinical workflow for clinicians to see during patient management, for example, titration of home medications based on notifications of outlier home BP values. This same workflow should be able to accommodate prescriptions of other PGD such as blood glucose, body weight, or oxygen saturation acquired by any clinically approved PGD device.

Current telemonitoring programs, however, often have a limited scope, address only 1 disease (eg, hypertension, diabetes, or heart failure), acquire only 1 type of remote data [2,3] (eg, BP or blood glucose), and support only a limited number of PGD devices (in terms of brand/model). This restrictiveness is at odds with the current technical capabilities of internet services in which data can be exchanged with a device-agnostic approach [4,5]. Email is a familiar example. Underlying standards permit email to be sent and read regardless of service provider, app, browser, or device used [6]. The current state of PGD-to-clinic integration lacks the seamlessness of email. Instead, health care organizations build and maintain custom connections with each device and their proprietary data formats. Such connections account for a large share of the cost of using PGD devices in clinical care [7], which constitutes a barrier to PGD usage [8].

This viewpoint formulates the integration of PGD into clinical systems and workflow as a *PGD integration pipeline* and reviews the functional components of such pipeline. We contrast the current state of integration to a standards-based pipeline using an example of integrating wireless BP data into primary care. We emphasize throughout the central importance of data standards in facilitating device-agnostic approaches needed to accommodate the complexity, scale, and rapid evolution of today’s health care systems.

## Standardized PGD Integration Pipeline

### Overview

Building custom connections between individual devices and health care organizations is costly and introduces data management inefficiencies. For organizations interested in remotely monitoring multiple types of health data via different PGD devices, one approach is to select 1 or a few device vendors for each data type and develop custom connections for each device to the electronic health record (EHR) [9]. Not only is this approach redundant, costly, and maintenance heavy, the dependence on vendor- or device-specific custom connections reduces flexibility to add or substitute new devices in the future.

**Figure 1:**
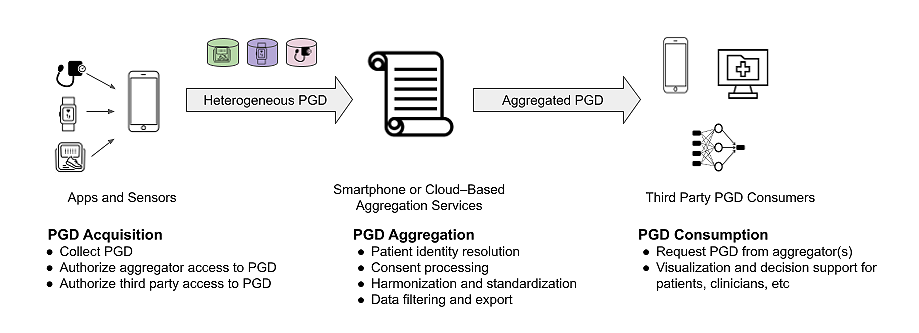
The person-generated data (PGD) integration pipeline comprises 3 components: PGD acquisition, aggregation, and consumption.

We can identify opportunities for streamlining the pipeline if we segment the 3 major functional components of PGD integration:

1. PGD acquisition: this encompasses PGD devices that manage person-facing functions such as consent and data collection;
2. PGD aggregation: this service manages consent, authentication, and authorization; maps data to standardized format(s); provides storage; and a query endpoint for third parties;
3. PGD consumption: third parties including EHRs, decision support systems, and analytic services provide applications that consume PGD to serve users such as clinicians and patients (Figure 1).

Currently, each PGD device manages its own acquisition, storage, and data usage, while each health care organization acts as a third party to multiple query endpoints, with each requiring their own integration into clinical workflow. Data standards would enable PGD from multiple devices to flow through a single pipeline instead of multiple pipelines, with each serving 1 device.

### PGD Acquisition and Data Sharing Consent

PGD are acquired from patients via a diverse and growing ecosystem of health tracking apps, wearables, and sensors [10]. Typically, a device will require a patient to download a smartphone app to establish an account and pull data from the device to the smartphone through Bluetooth to store on the device company’s cloud. Many devices provide an app or online dashboard where a patient or their physician can view tracked data [11-13].

Once data are acquired by the device, patients provide consent for data sharing either directly or via a separate PGD aggregator app that will serve the data to third-party solutions and their users (Figure 1). Existing aggregator apps include Apple Health, Google Fit, CommonHealth, Human API, and Validic. Apple Health and Google Fit allow patients to further share their data with any participating third party in the iOS and Android ecosystem, respectively, but with somewhat opaque rules by which third parties can request data and without any evaluation of clinical validity or security. CommonHealth, a nonprofit entrant to the personal health record/aggregator app space, differs by establishing a Common Trust Framework [14] in which patients can consent to share downloaded EHR or device data with trusted apps and services running on their phone. This framework is a neutral, independent set of rules that is developed through open-community governance.

Data-sharing consent can be granted at different levels of granularity. Patients may authorize their clinician to access only their BP data, while authorizing a clinical trial they are enrolled in access to BP, step count, weight, and calorie tracking data. Consent may also be revoked entirely or temporarily withheld for privacy or other reasons (eg, withholding weight data while on vacation).

### PGD Aggregation

Once patients consent to data sharing, an aggregator app’s service processes their consent to mediate data transfer. PGD aggregation service components include authenticating third-party data requests, resolving whose data are being requested, managing authorization and consent, securely storing data (if needed), mapping data to standardized format(s), and exporting data in the desired standardized format to the third party.

### Authentication, Authorization, Identity Resolution, and Consent Management Handling

#### General Practice

Standard industry procedures such as OAuth2 [15] are used for delegated authorization between PGD aggregator and third parties. Delegated authorization allows patients to authorize different services to access their data without services needing to expose personal credential information to each other. However, identity resolution between multiple services is challenging as it is common for patients to have several health care accounts (eg, their clinic, laboratory, and pharmacy).

Identity resolution within health care accounts, such as EHR services, is mediated via patient Fast Healthcare Interoperability Resources (FHIR) IDs. However, a patient will have different FHIR IDs for every health organization they access, and an organization may have multiple FHIR IDs for each patient depending on the back end implementation of FHIR servers. Without a national unique patient identifier, PGD aggregators will have to approximate patient identity. Linking many FHIR IDs and devices with apps requires tedious combinations of authorization flows subsequently further complicating consent management, as the PGD aggregator must match a patient’s data-sharing consent against any third party requests for the patient’s data. The complexity of consent management architectures argues strongly for standardized reusable multipurpose PGD integration pipelines.

#### Data Storage

The PGD aggregator can either pass-through or store-and-forward data depending on the business need. With pass-through, the aggregator ingests data from the phone or device cloud and sends them directly to a third party at each request. With store-and-forward, the aggregator persists the data. Benefits of the pass-through approach include lower costs and security risks because the aggregator does not store data. Downsides include increased latency in data access, inability to perform computations (eg, average of requested values), and the need to repeat any mapping to standardized data formats. In a store-and-forward model, data can be persisted in native or in any standardized format.

PGD aggregators often have an on-phone and a cloud component. Some are PGD only (eg, Google Fit) while others (eg, Apple Health, CommonHealth) also aggregate EHR data. Apple Health and CommonHealth keep all synced data on the patient’s smartphone; Google Fit uploads the data to Google Cloud.

#### Standardized Data and Metadata Export

**Figure 2:**
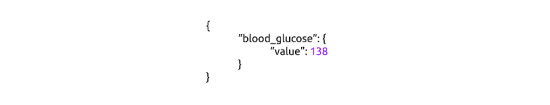
This figure shows a JSON instance of a blood glucose value of 138. No other data or metadata are available.

**Figure 3:**
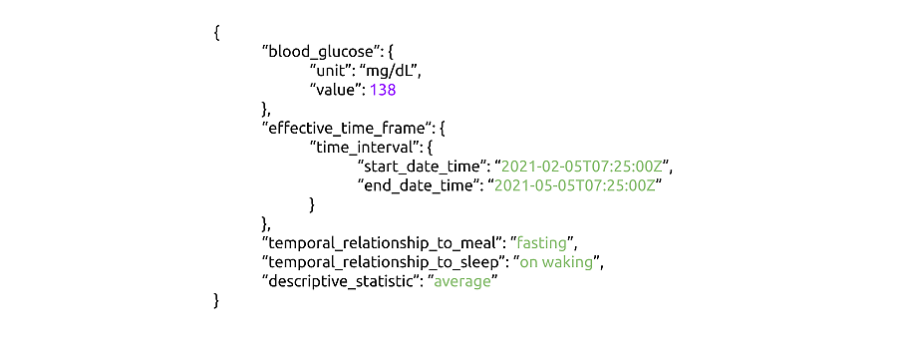
This figure shows an Open mHealth–compliant JSON instance of blood glucose with metadata showing that the value of 138 mg/dL is the average fasting value on awakening between February 5 and May 5, 2021.

Most existing aggregators export PGD to third parties using their own nonstandardized formats [16-18]. CommonHealth, by contrast, exports data in standardized formats: EHR data are exported in Health Level 7 (HL7) FHIR format and PGD in Open mHealth/IEEE 1752.1 format. This difference is crucial. Clinically relevant contextual information is necessary for making clinical decisions. As shown in Figure 2, a blood glucose data of “138” is clinically meaningless unless the units, any relationship to meals or sleep, and effective time (ie, when the observation applied in the real world, not when the value was reported) are made clear. Standardized selection, definition, and value sets, as in Figure 3, for these contextual variables (eg, Unified Code for Units of Measure [UCUM] for units) would allow third-party systems to reliably and unambiguously understand the meaning of the PGD value, a minimal requirement for using PGD in health care or research.

In addition to clinically relevant contextual information, use of PGD in health care or research also requires metadata [19,20]—data about the data. Examples include the name, model, and unique ID of the source device, and the unique ID of the app [21] installed on the patient’s smartphone that acquired the data. Table 1 lists examples of metadata of interest for a sleep digital biomarker.

While there is no end to the types of metadata that would be of interest to someone for some purpose, it is infeasible to collect all possible metadata on all PGD. Nevertheless, a minimal set of critical metadata must be available on all PGD values to enable ecosystem-wide quality assurance, auditing, and regulatory oversight. The PGD ecosystem must therefore coalesce around a core set of data and metadata standards to enable long-term integrity and usability of PGD. Data can be standardized at the point of export by PGD devices or PGD aggregators can harmonize and provide endpoints for standardized PGD. Table 2 lists the standards that are most relevant to PGD. At the device level, standards such as IEEE 11073 [22] and FHIR’s device resource [23] address manufacturing, security, privacy, and data export issues. For PGD integration, the Open mHealth/IEEE 1752.1.1 standard is the most directly relevant, covering the most widely used PGD variables for sleep, physical activity, cardiovascular, and other domains with over 80 JSON schemas [24,25]. Value sets are standardized using terms from Systematized Nomenclature of Medicine (SNOMED) or Logical Observation Identifiers Names and Codes (LOINC). A minimal metadata schema is used for the JSON schema header with standardized pointers to externally held metadata information (eg, an UDI registry). Open mHealth schemas are open source, free to all, and are the output of a global community of stakeholders consisting of developers, data scientists, informaticians, researchers, and clinicians. The sleep, physical activity, metadata, and utility schemas (on units, time, etc.) comprise the global standard IEEE 1752.1.1 [25].

**Table 1:** Metadata of a sleep digital biomarker.

Metadata category	Example questions
What is the biomarker about?	Sleep duration? Sleep quality? Sleep refreshment?
Definition (eg, for total sleep duration)	Time in bed? Time asleep? With or without micro awakenings?
Validity	How does the biomarker compare with a gold standard?
Error	How much does it vary from the gold-standard value?
Natural variability	What is the natural variability within and among individuals, for comparison to the error range?
Uncertainty/Confidence	What is the probability that the person was asleep during this time?
Bias	Are there systematic errors in different populations?
Identity	Was the measurement collected for the right person?
Context	Was there relevant contextual information? For example, at home versus on a trip across time zones.

**Table 2:** Selected standards relevant to mobile health.

Standard	Description
HL7^a^ FHIR^b^	HL7 refers to a set of international standards for transferring clinical and administrative data between health care providers. Within HL7, FHIR describes the data schema and application program interface for exchanging EHR^c^ data.
IEEE 11073	A family of standards for medical device communication, including point-of-care clinical devices and personal health devices.
IEEE 1752	A family of standards for representation of person-generated health data, based on work by Open mHealth.
CTA^d^	A set of standards specifying how products work and the ways consumers interact with them. A subset of the standards pertain to consumer technologies in the health and fitness space [26].

^a^HL7: Health Level 7.

^b^FHIR: Fast Healthcare Interoperability Resources.

^c^EHR: electronic health record.

^d^CTA: Consumer Technology Association.

### PGD Consumption

Third-party users occupying the distal end of the PGD integration pipeline include health care organizations, researchers, and patients themselves (eg, consumers of an app that provides predictive analytics for blood glucose control). Many third parties want to be device agnostic. For example, a company providing decision support for BP management would want to accept BP data from any FDA-cleared brand and model of wireless BP cuff. Many third parties may also need to integrate heterogeneous data sources, such as reconciling sleep data from a smartwatch and a dedicated sleep sensor. Third parties would enjoy great efficiencies if PGD were available in a common data and metadata standard by not needing to divine the contextual meaning or metadata of PGD acquired from different sources. A standardized endpoint from a PGD aggregator would support the ideal of collecting PGD once and reusing them for multiple purposes.

## Illustrative Case

### Home Blood Pressure Integration

Home BP monitoring (HBPM) programs, in which dedicated staff monitor the home BPs of a panel of patients with hypertension for treatment support and adjustment, have shown efficacy in improving BP control [27]. Health care organizations are thus increasingly interested in establishing HBPM programs [8], which are reimbursable under several Centers for Medicare & Medicaid Services (CMS) billing codes but only if home BP measurements are acquired via wireless-connected cuffs and written directly into the health care organization’s EHR [28]. We illustrate the PGD Integration Pipeline using the example of integrating wireless BP data into an EHR.

### Current Status: Home Blood Pressure Integration

Currently, home BPs from connected devices can be brought into an HBPM program through several pathways. One pathway is for patients to manually enter home BPs into an EHR patient portal. Despite its simplicity, this approach has many downsides. Using patient portals is challenging for patients with language barriers and low technology skills [29]. Manual reporting may result in fewer datapoints, is difficult to sustain over time [30], and evaluation and management of manually reported BP data are not reimbursable by CMS under the remote physiologic monitoring codes [28].

Another approach involves a partnership between a health care organization and a single wireless BP cuff company which will offer that company’s online dashboard for clinicians to view. The need for clinicians to login to the company’s website outside of their EHR severely disrupts workflow and is usually vehemently opposed by clinicians. Moreover, to qualify for CMS reimbursement, a custom interface has to be built and maintained to write data from that company into the EHR. Not only is this time-consuming and expensive, but it also severely limits flexibility. Adding another brand of cuff would require an entirely new integration effort and online dashboard. Inertia to stay only with the initial company would be high. Such “vendor-lock” is inadvisable in a fast-changing digital health world.

An emerging approach takes advantage of Apple Health and Google Fit as PGD aggregators. At University of California, San Francisco (UCSF), which is on the Epic system, a pilot project is allowing clinicians to prescribe HBPM and have that prescription displayed on their patients’ MyChart portal. Patients use one of several brands of cuffs, download both the device’s app and the MyChart app onto their smartphone, and use Apple Health or Google Fit to consent and direct their BP data from the device’s app into Epic. The ingested data can then be viewed within Epic and evaluation and management can be billed under CMS codes. This approach has the benefits of being device-agnostic, billable, and integrated into the EHR-based workflow. However, it is reliant on, and constrained by, Epic, MyChart, Apple Health, and Google Fit functionality and usability. Indeed, as of this writing, Google Fit has “temporarily stopped” accepting connected BP values and other “sensitive” health data types including body temperature and oxygen saturation [31]. Moreover, using PGD aggregators without data standards–driven infrastructure impairs robust PGD validation and use. For example, device manufacturer data are unavailable for query from either Apple Health or Google Fit endpoints.

### The Goal: Standardized Home Blood Pressure Integration

The optimal approach of using data standards throughout the BP integration pipeline offers many benefits. First, if device vendors adhered to data and metadata standards (eg, Open mHealth/IEEE 1752.1.1), the meaning and context of BP and other PGD would be captured for posterity at the source, which is ideal for downstream use, auditing, and regulatory oversight. If BP data are not standardized at the source, a standards-based PGD aggregator such as CommonHealth can ingest and map BP data from multiple vendors into Open mHealth or FHIR for standardized export. Health care organizations using a standards-based PGD aggregator are ensured that BP data will come in a consistent format with the same clinical contextual information and metadata, regardless of the cuff’s brand or model. This device-agnostic predictability within a single integration pipeline yields great flexibility: multiple types of PGD from different device vendors can be integrated.

For health care organizations, standardization can facilitate data integration into workflow and writing into the EHR for billing. EHRs in the United States must now by law support HL7 FHIR data and protocol standards [32]. This allows EHRs to receive and display PGD using SMART-on-FHIR [33] protocols to launch dashboards directly in the EHR without requiring separate login. The mPROVE project at UCSF is taking this approach [34], displaying patient-reported outcomes and BP data in the BRIDGE SMART-on-FHIR dashboard [35]. Using SMART-on-FHIR frees data display and decision support presentation from the constraints of the EHR. While still in early adoption, SMART-on-FHIR technology has tremendous promise to augment the distal end of the PGD integration pipeline.

Another valuable benefit of data standardization for health care organizations is increased efficiency of data integration [36]. Instead of having to build and maintain custom connections to multiple device vendors, an organization receiving PGD in a common predictable format such as Open mHealth/IEEE 1752.1.1 can reuse the same interface for bringing PGD into their EHR or clinical workflow. Going forward, this singular interface can accommodate any new PGD data type that is supported by the data standard. The organization can flexibly switch to any other PGD aggregator that supports the same data standard because the PGD remains consistent for interfacing into the EHR.

Finally, the promise of PGD will be realized only if patients trust how their PGD will be handled, and if collecting, consenting, understanding, and sharing PGD are sufficiently easy to do [37]. To the extent that standardization of the PGD integration pipeline reduces data silos, multiple identities and accounts, and a profusion of opaque data-sharing mechanisms, trust will be enhanced for all parties and PGD integrity and value will be increased.

## Discussion

### Highlights

Today’s mHealth data ecosystem—where multiple apps, devices, and proprietary aggregators each export data in their own data formats with little context or metadata—is suboptimal for unleashing the full capabilities of mHealth technologies to improve clinical care. Standards are key to successful data interchangeability and should be adopted broadly to enable device-agnostic solutions and modularity and to simplify the PGD ecosystem while simultaneously supporting data validation and data integrity.

### Relationship to Digital Biomarker Validation and App Frameworks

Deployment of PGD solutions in clinical care needs to extend beyond interoperability and integration. Various frameworks and best practices exist for choosing and deploying mHealth apps and sensors. HL7’s Consumer Mobile Health Applications Functional Framework (cMHAFF) provides industry guidance and common methods to assess the “foundational characteristics,” including but not limited to security, privacy, data access, data export, and transparency/disclosure of conditions, of mHealth apps [38]. HL7’s App Data Exchange (ADE) project documents the functional requirements and provides a framework supporting data exchange between mHealth devices, apps, and other parts of the health IT Infrastructure [39]. The ADE project references mHealth data standards such as Open mHealth/IEEE 1752.1.1 and IEEE 11073.

By themselves, neither cMHAFF nor ADE address the clinical validity or value of an mHealth solution. The DiMe Playbook is a “comprehensive ‘how-to’ guide” on developing, selecting, and deploying digital biomarkers. It addresses digital biomarker verification, analytical validation, and clinical validation (V3) as well as the role of standards such as Open mHealth/IEEE 1752.1.1 in data integration [40].

### Toward Interoperability by Design

Like privacy, data provenance and interoperability should be intentionally designed into a system up-front rather than shoehorned into it later on [41]. For the mHealth ecosystem, a mix of frameworks, official data standards and protocols, and best practices as reviewed above is beginning to paint a path out of today’s fragmented silos. For the PGD integration pipeline in particular, the path includes PGD devices and mHealth apps exporting and consuming digital biomarkers in the Open mHealth/IEEE 1752.1 format where appropriate; expanding the data types standardized by Open mHealth/IEEE 1752.1; data aggregators exporting PGD in both their current format (to ensure backward compatibility) and Open mHealth/IEEE 1752.1 format (to transition toward standardized interoperability); and finally, wide adoption of the Open mHealth-to-FHIR implementation guide as the common FHIR observation resource profile for PGD [42]. These steps offer a glidepath for the ecosystem to transition to data and metadata standards that themselves evolve to accommodate new digital biomarkers and new metadata frameworks. Further research is needed on scalable metadata acquisition and management, biomarker validation platforms, and interoperability with the broader internet of things.

### Conclusion

The clinical value of PGD from mHealth apps and sensors is currently limited by difficult and inefficient integration into routine clinical care. Major components of the PGD integration pipeline include PGD acquisition, PGD aggregation, and third-party solutions that consume PGD to deliver end value for clinical care and clinical research, all while retaining people’s control on their data and trust in the process. Standardization of data and metadata along the entire PGD integration pipeline is crucial for ensuring device-agnostic, modular, flexible, multipurpose, and thus lower-cost integration into clinical workflow. The value of efficient integration of PGD data will increase revenue streams, reduce overhead, improve data integrity, and facilitate patient trust. PGD aggregation services that offer standards-based PGD integration play a vital role in transitioning from today’s siloed friction-heavy data ecosystem to a low-friction interoperating system that our patients deserve. Health leaders responsible for remote monitoring and other PGD programs should seek out and adopt pipeline-based approaches to standardize the integration of PGD into clinical care.

## Abbreviations

ADE: App Data Exchange
BP: blood pressure
cMHAFF: Consumer Mobile Health Applications Functional Framework
CMS: Center for Medicare and Medicaid Services
EHR: electronic health record
FHIR: Fast Healthcare Interoperability Resources
HBPM: home blood pressure monitoring
HL7: Health Level 7
LOINC: Logical Observation Identifiers Names and Codes
PGD: person-generated data
SNOMED: Systematized Nomenclature of Medicine
UCSF: University of California, San Francisco
UCUM: Unified Code for Units of Measure
